# The prevalence of mental distress and the association with education: a cross-sectional study of 18-49-year-old citizens of Yangon Region, Myanmar

**DOI:** 10.1186/s12889-020-8209-8

**Published:** 2020-01-22

**Authors:** Win Thuzar Aye, Lars Lien, Hein Stigum, Hla Hla Win, Tin Oo, Espen Bjertness

**Affiliations:** 1 Department of Preventive and Social Medicine, University of Medicine, Yangon, Myanmar; 2 Department of Community Medicine and Global Health, Institute of Health and Society, University of Oslo, Oslo, Norway; 3 National Norwegian advisory board for concurrent addiction and mental health problems. Innlandet Hospital Trust, Brumunddal and Faculty of Social and Health Sciences, Inland Norway University of Applied science, Elverum, Norway; 4 University of Public Health, Yangon, Myanmar; 5 Department of Mental Health, University of Medicine 1, Yangon, Myanmar

**Keywords:** Prevalence, Mental distress, Myanmar

## Abstract

**Background:**

Poor mental health is an important contributor to the global burden of disease. Mental health problems are often neglected in communities, and are scarcely studied in developing countries, including Myanmar. This study estimates the prevalence of mental distress by socio-demographic and health related factors, and the association between education and mental distress. As far as the authors are aware, this is the first population-based study in Myanmar estimating the prevalence of mental distress.

**Methods:**

Between October and November 2016, a cross sectional study was conducted using a multi-stage sampling design with face-to-face interviews using the Hopkins Symptom Checklist (HSCL-10) for mental distress (symptoms of depression and anxiety). The multivariable analysis strategy was based on Directed Acyclic Graphs (DAGs), to identify confounders, mediators and colliders. Pearson’s chi-square was used for testing differences between proportions and multiple linear regression analysis was applied to explore the association between education (years at school) and mental distress (HSCL score).

**Results:**

A random sample of 2391 (99.6% response) men and women aged 18–49 years participated in the study. The prevalence of mental distress was 18.0% (95% confidence interval (CI): 14.7–21.9), being higher among women (21.2%; 95% (CI): 16.6–26.6) than men (14.9%; 95% (CI): 11.4–19.2). Older-age, being separated or divorced and having a higher number of children were associated with increased mental distress. In linear regression analyses, adjusted for confounders (age, marital status and income), there was a significant negative association between years at school and mental distress among women and older men (> 30 years), but not among the youngest men.

**Conclusions:**

The prevalence of mental distress is high, and there is an association between HSCL-10 score and education. Due to the scarcity of mental health services in Myanmar, the findings indicate a need for a mental health policy to handle the burden of mental health problems in Yangon, a burden which is probably high within the country.

## Background

Mental distress or common mental health problems are two terms used synonymously [1]. They are defined as an emotional state of health, characterized by symptoms of depression and/or anxiety [2]. These symptoms result from exposures to events that individuals are not able to cope with, and can further lead to a depression or anxiety disorders (common mental disorders) [2, 3].

The burden of mental health problems in low and middle-income countries (LMIC) is high [4]. Based on the global burden of disease (GBD) study in 2017, mental health problems accounted globally for 19.3% of years lived with disability (YLDs) and 9.4% of disability-adjusted life-years (DALYs) in adults (15–49 years) [5]. In the South East Asia region, mental health problems contributed to 18.6% of YLDs, while in Myanmar the contribution was 14.2% [5]. Furthermore, the common mental health problems (mental distress: i.e. depression and anxiety symptoms) are among the top ten leading causes of DALYs in Myanmar [5]. A contributing factor to the burden of mental distress in Myanmar could be related to circumstances when Myanmar was under a military government for more than 50 years, ending in 2011. The country faced many socio-political difficulties during this time-period [6] which may have affected people’s wellbeing. Natural disasters, economic crisis, violence, wars and migration [6] may have reinforced anxiety and depression symptoms. Another contributing factor is that mental health problems tend to occur concomitant with chronic diseases like cancer, musculoskeletal disorders, coronary heart disease and diabetes [7, 8]. The increasing burden of these diseases in Myanmar [5] might have contributed to the mental health situation. Moreover, there is low awareness about mental health problems in Myanmar [9, 10]. Many people still believe that evil spirits and witchcraft cause mental health problems [10]. Stigmatization and discrimination from society has resulted in mental health problems becoming a hidden epidemic [9, 10].

Population-based studies among adult populations in low-and middle-income countries and high-income countries have estimated the prevalence of common mental health problems in Cambodia 27% [11], Vietnam 14.2% [12], Thailand 9.3% [13], 29.2% Malaysia [14], Canada 38% [15] and 11.4% Norway [16]. In Myanmar, population-based studies on the prevalence of mental distress are lacking. There is, however, a small-scale survey conducted in 2004 in a peri-urban and rural area of Myanmar, which estimated the prevalence of anxiety and depressive disorders to be 4.1 and 0.5%, respectively [10], however further information regarding instruments used and sample size was not reported.

Recently, the Millennium Development Goals (MDGs) were replaced by a new era of the Sustainable Development Goals (SDGs) that included mental health under Goal 3, target 3.4, to reduce by one-third premature mortality from non-communicable diseases (NCDs) through prevention, treatment and promotion of mental health and wellbeing. Thus, the SDGs cannot be achieved without the inclusion of mental health [17], and it could be useful to view mental health policies in the perspective of social determinants of health [3, 18]. The World Health Organization (WHO) points out that social determinants of health impact on individuals health throughout the life cycle, in which “people are born, grow, live, work and age” [18]. A recent report on Social Epidemiology and Global Mental Health indicated that main social determinants such as gender, poverty, education, marital status and ethnicity were significantly associated with mental health problems [4]. Likewise, several other studies revealed that social inequality impact an individual’s mental health [11–15]. Also, the report by WHO and Calouste Gulbenkian Foundation emphasized that social determinants of health should be addressed in the planning programs for prevention and promotion of mental distress in the community [3]. Of the various social determinants of mental health, education is one of the integrated parts of social, cultural and economic development and has a great impact on a person’s health. Higher level of education is linked to employment, higher income, higher social status and good health seeking behavior [19, 20]. On the other hand, some studies suggested that higher education leads to stressful events early in life and increases the risk of future episodes of mental distress in adult life [21–23].

The objectives of the present study among 18–49 year old citizens of the Yangon Region in Myanmar are to estimate the prevalence of mental distress by selected socio-demographic and health related factors, and to estimate the association between education (years at school) and mental distress among men and women.

## Methods

### Study design, sampling and population

A population-based cross-sectional survey was conducted from October to November 2016 in the Yangon Region; the most developed and densely populated area of Myanmar. The population of the Yangon Region is 7.4 million, where 5.2 million reside in urban areas and 2.2 million reside in rural areas [24]. It accounts for 14.3% of the entire Myanmar population [24]. The Yangon Region is composed of four districts, of which only the North and South include both, urban and rural populations, and the East and West are urban only. Therefore, the North and South districts were purposively selected for this study.

**Fig. 1:**
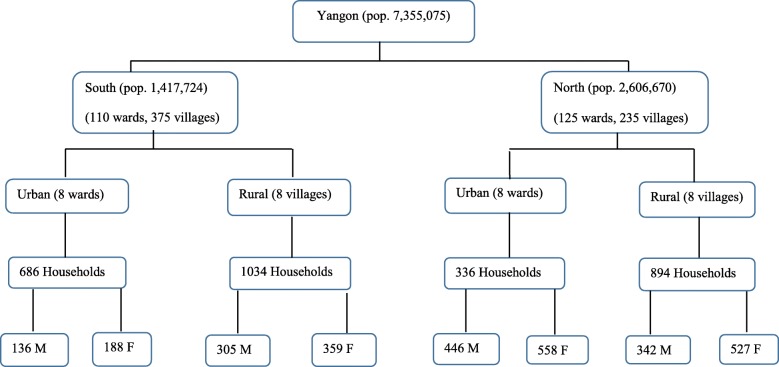
Sampling flow diagram

We applied a multistage study design. The sampling frame for each district was based on the 2014 census [24], with villages (in rural areas) and wards (in urban areas) used as accounting areas. A “ward” is a segment of an urban township. In the first step of sampling, we selected North and South districts. The second step was to select urban wards and rural villages from these two districts. There are 125 wards and 235 villages in the Northern district and 110 wards and 375 villages in the Southern district. Eight wards and eight villages were randomly chosen from each of the two districts, i.e. a total of 16 wards and 16 villages. In the third step, households were randomly selected from each ward and village. The number of households selected in the two districts was based on the population size of the districts and by the urban–rural distribution, using proportional probability sampling. The population distribution is 64.8 and 35.2% in the North and South district, respectively [24]. We randomly selected one woman and one man from every other household based on a list of households with at least one person aged 18–49 years old. We obtained information from the District Health Department in Yangon Region about the number of wards and villages in the selected districts. The number of men and women aged 18–49 years old in the selected wards and villages were obtained with the help of the local authorities, midwives or lady health visitors. In each household, one person was asked to list all of the family members within the age range of 18–49. From this list, one person was randomly invited to participate in the study using the sealed envelope method [25]. In total, 2400 invitees were identified, and 2391 (99.6%) participated in the present study, 1547 from the Northern district (889 from urban and 658 from rural) and 844 from the Southern district (254 from urban and 590 from rural) (Fig. 1).

Both men and women aged 18–49 years, independent of ethnicity, were invited to the study. The age group of young adults up to middle age was chosen, as the present study is a part of a larger project targeting women in reproductive age. We excluded people who were not ordinary residents such as military personnel, Buddhist monks and nuns and other institutionalized persons, and those who were physically or mentally too ill to participate. The sample size was calculated based on the expected prevalence of exposure to domestic violence among women committed by the spouse (21%), previously reported in the 2016 Demography and Health Survey (DHS, 2015–16) [26]. The prevalence of domestic violence was used in the sample size calculation because it is a main outcome in the larger project, in which the present study is a part of.

### Data collection

Data was collected from structured interviews, a modified version of the Myanmar Demography and Health Survey (DHS) [26], which was already translated to Burmese. The Hopkins Symptom Checklist (HSCL-10) [16] was included for recording of mental distress, which was translated from English to Burmese by a psychiatrist and back translated to English by an English professional.

The principal investigator and 12 trained field workers (MDs) carried out the data collection. Both male and female interviewers with experience in population-based surveys were recruited. A two-day course was conducted for the purpose of training in interviewing techniques, to learn about the purpose of the study, sampling methods, interpersonal communication skills, informed consent, and the survey questionnaire.

A pilot survey was conducted on the 1st and 2nd of October 2016 in 54 households of one Ward of the Dagon Seikkan township, a township not included in the survey sample. During this survey, interviews were conducted in order to assess clarity, cultural acceptability and understanding of the questions. Based on these results, a minor amendment was created in the Burmese version of the HSCL-10.

Male fieldworkers interviewed male respondents and female fieldworkers interviewed female respondents. After selecting the eligible person, the interviewer obtained informed consent to undertake the survey at the onset of the interview. In order to ensure privacy, a separate room or area outside of the household was chosen before the interview of the respondent. In the event of the respondent being out of reach at the time of the initial visit, the interviewer made at least two repeat visits on that same day. Due to resource limitations and travel distances, it was decided not to return to the household on another day. In the case of no one being present at the household on the day of the interview, or if the participant did not want to participate, they were regarded as non-responders. If a respondent became distressed during the interview, the interview was stopped, and only continued if the respondent was willing, and able to continue answering the questions. After collecting the data from the interviewer, the supervisor checked the completeness of each questionnaire. When answers were missing (non-refusal), a return visit to the household was made and the questions were asked again. Data entry was completed using Epidata software, version 3.1.

### Study variables

Mental distress was assessed using The Hopkins Symptom Check List-10 (HSCL-10), a mental health-screening tool that emphasizes the dimensions of depression and anxiety. This tool is recommended for screening purposes because the instrument has high sensitivity and specificity in identifying ‘non-distressed’ and ‘distressed’ groups in the general population [16]. It was developed from HSCL-25 [27] and consists of 10 items on a four-point scale ranging from 1 (not at all) to 4 (extreme) with a higher mean score indicating increased mental distress. The 10 items included in this shorter version are; feeling panicky, anxious, dizzy, tense, sleepless, sad, worthless, hopeless, fault within self and finding everything a burden. The presence of symptoms during the past week (including the day of the interview) was recorded, of which six items are related to depression and four items are related to anxiety. The reliability and validity of HSCL-10 as a screening measure for mental distress has been demonstrated in community surveys in Norway [16] and Pakistan [28]. In the present study, the internal consistency (Cronbach α: .85) was similar to the Pakistan population-based study (.86) [28]. According to Strand et al. [16] and Sørlie et al. [29], the cut-off criteria of 1.85 is significantly associated with an increased risk of depression and anxiety (mental distress).

Socio-demographic variables collected during the survey included age, gender, location, marital status, family members, educational level, occupational status, income, number of children, migration and household debt. Age (years) of the respondents was categorized into age-groups of 18–29, 30–39 and 40–49. For educational levels, we operationalized the number of years at school into three separate groups: ≤ 5 years (also includes those with no formal schooling); 6–11 years; and more than 11 years of schooling. Age and education (years of schooling) were also used as a continuous variable in multivariable analysis.

With regard to household income, the total monthly household income was divided by the number of residents regardless of age, generating a per capita monthly income. Daily individual income was categorized into three groups according to the World Bank’s cut-off of poverty lines of 1.90 USD/day and 3.10 USD/day [30] i.e. low (≤ 1.90 USD/day), medium (between 1.90–3.10 USD/day) and high (≥ 3.10 USD/day).

Occupational status was divided into dependent, unskilled worker, government staff, non- government staff and small business owners. Unskilled workers included individuals undertaking odd jobs, dependents (including students), unable to work, and housewives. Marital status was categorized into currently married, never married (single), separated, divorced or widowed.

Health status variables: current health status was self-reported based on the question “In general, how would you characterize your current health?” The response options were poor, not very good, good and very good. The responses were operationalized into “poor health” (poor, not very good) and “good health” (good, very good). To determine functional disability, the question “Do you have impairments …” was asked, with possible replies being “no”, “yes, a little”, or “yes, a lot”. With regard to mobility, vision, hearing, the ability to bathe one’s self, dressing and remembering or concentrating, the responses were categorized into “yes” and “no” [31].

Muscle and skeletal pain were measured by the following questions: “Have you in the last 12 months experienced pain several times in: head, neck/shoulders, arms/legs/knees, stomach, and back?” with responses being “yes” or “no”. Based on the answers given, three groups were created: 0 pain sites, 1–2 pain sites and 3 to 5 pain sites [32].

Domestic violence (DV) was recorded if the respondent was exposed to physical, sexual and/or emotional violence committed by the intimate partner (for married / partnered participants only), or if the respondent, after the age of 15 years, was exposed to physical violence committed by anyone else (for all participants), or if the respondent was exposed to childhood sexual violence (for all participants). The domestic violence questions were obtained from the WHO Myanmar Demographic and Health Survey [26].

### Data analysis

STATA/IC version 15.0 was used in the data analysis. We used the survey prefix command “svy” for complex survey data. For descriptive analyses, we estimated prevalence with 95% confidence interval (CI), mean with standard deviation (SD) and median values with Inter Quartile Range (IQR). For bivariate analysis, a Pearson’s chi-square test was used for testing differences between proportions. Multiple linear regression was used to estimate the association between years at school (education) and HSCL score (mental distress), with separate analysis for men and women.

**Fig. 2:**
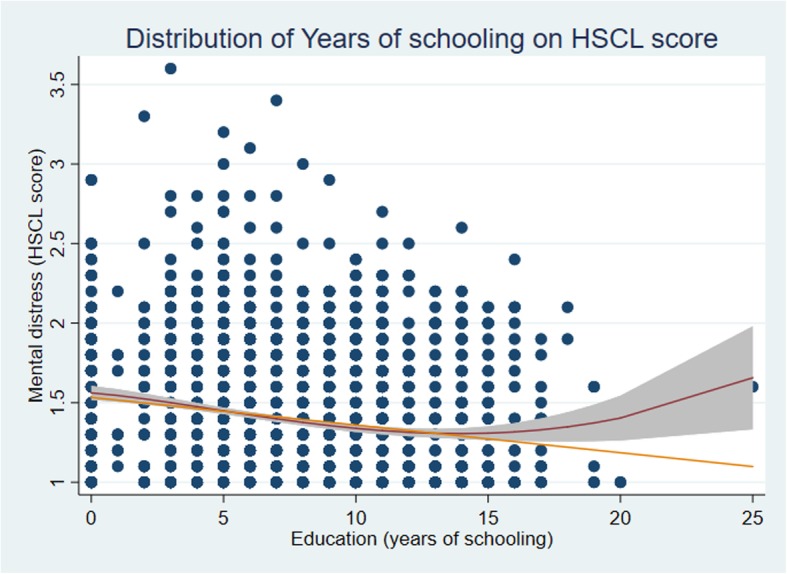
Distribution of HSCL scores by years at school among 18–49-year-old citizens of Yangon Region, Myanmar

**Fig. 3:**
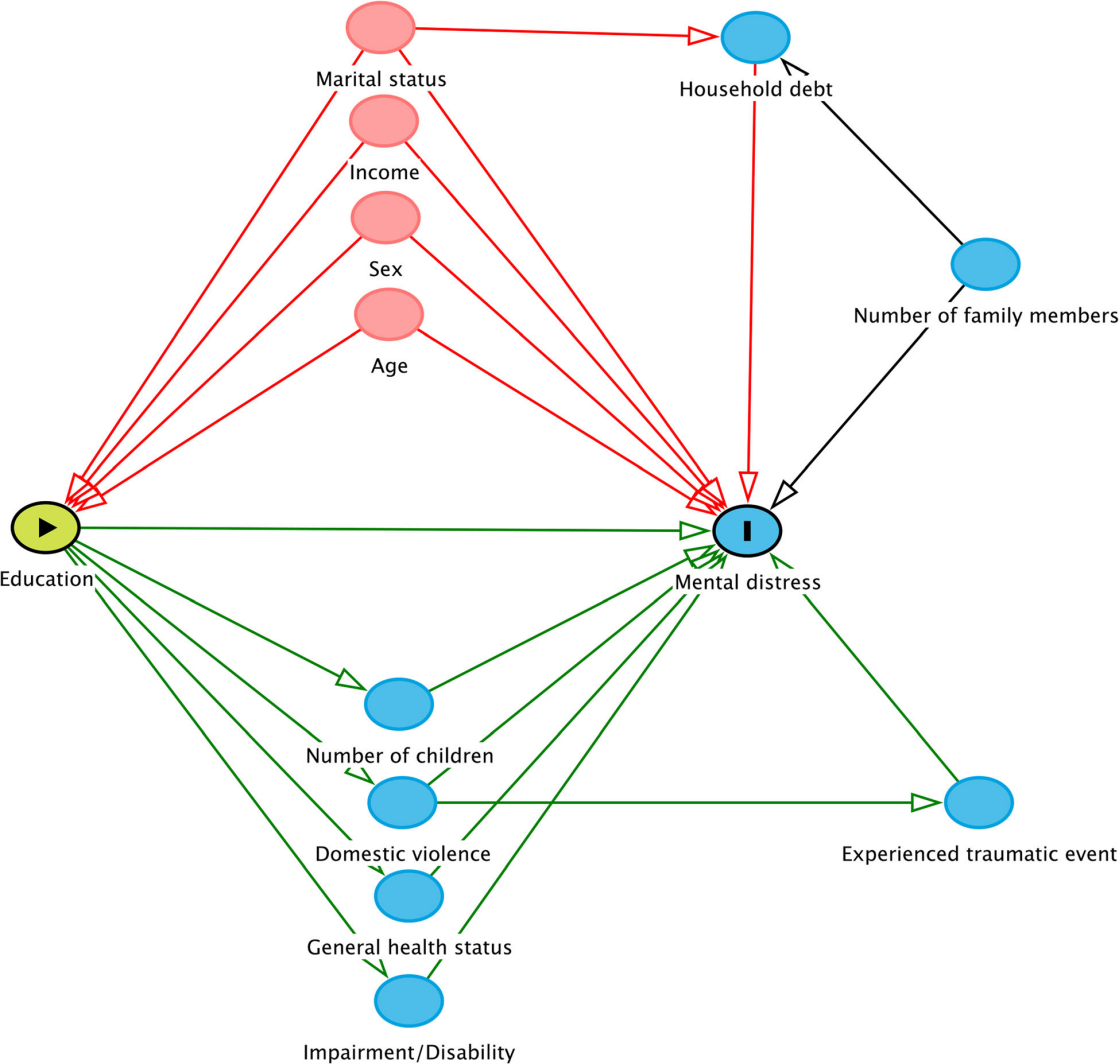
Directed Acyclic Graph for the association between education (years at school) and mental distress [33]

**Fig. 4:**
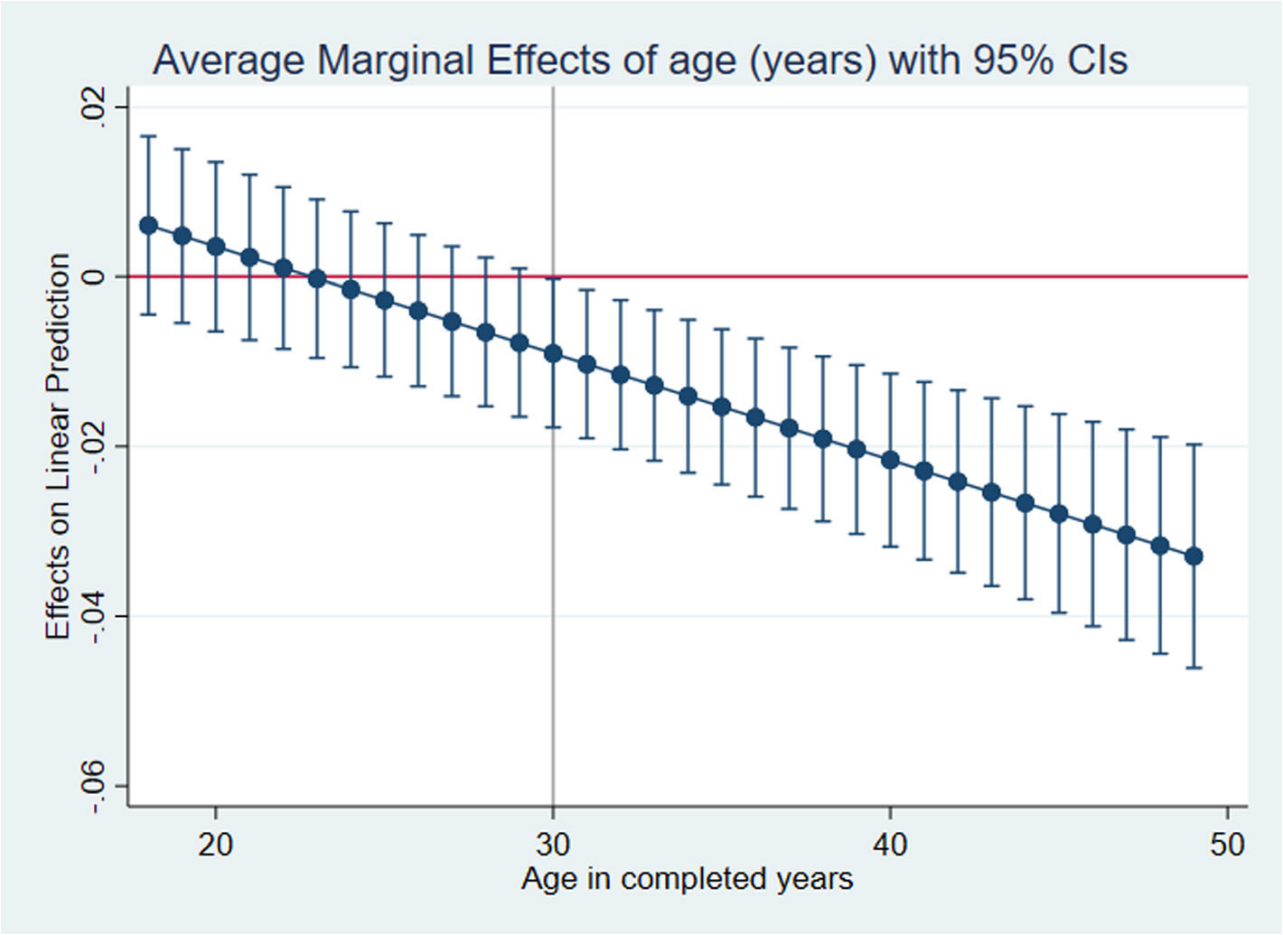
Marginal effects of education on mental distress in men 18–49-years-old of Yangon region, Myanmar

We checked the distribution of years at school by mental distress (HSCL score) (Fig. 2). We drew a directed acyclic graph (DAG) (Fig. 3) to illustrate our strategy of analyzing data (multivariable analyses). We identified confounders, mediators and colliders based on prior knowledge about the connection between exposure and outcome. We adjusted for cofounders, however not for mediators since we are interested in estimation of the total effect of the exposure. If the mediating variables are adjusted for in the model, that path will be closed and we no longer see the total effect of the exposure on the outcome. Moreover, that may cause over-adjustment bias in the estimation of the total effect and reduce precision [34]. Based on the DAG (Fig. 3), age, income and marital status were identified as confounders in order to estimate the total effect of education on the HSCL score (mental distress). We detected an interaction between education and age, which we adjusted for in the estimation of the total effect of education on mental distress. We analyzed the marginal effect of the association between years at school and mental distress at low age (18 years) and high age (49 years) in men, using a marginal plot analyses (Fig. 4). The assumptions of a linear model (linear effects and constant error variance) were tested by plotting residuals versus predicted values. We looked for observations with high influence by plotting delta-betas versus observed numbers. All significance tests were two-sided, and *p*-values < 0.05 were considered to be statistically significant.

## Results

We analyzed 2391 participants, of which 49.8% were women. The mean age was 33.1 ± 9.1 (SD) years. The prevalence of mental distress in the total sample was 18.0% (95% CI: 14.7–21.9) and mean HSCL score was 1.4 ± 0.4. The prevalence was significantly higher in women (21.2%; 95% CI: 16.6–26.6) than in men (14.9%; 95% CI: 11.4–19.2), and there was a significantly higher prevalence among residents in the urban population (21.7%; 95% CI: 17.0–27.2) compared to the rural population (15.0%; 95% CI: 12.1–18.6) (*p* = 0.03) (not shown in Table). Moreover, women in urban areas had a higher prevalence of mental distress compared to women in rural areas (Table 1).

**Table 1:** Prevalence of mental distress^a^ and median HSCL-10 score by socio-demographic and selected health-related factors among men and women 18–49-year-old citizens of Yangon Region, Myanmar

	Men (*N* = 1200)				Women (*N* = 1191)			
	N	Prevalence n (%)	p-value	Median (IQR)	N	Prevalence n (%)	p-value	Median (IQR)
Age group			0.004*				0.06	
18–29 years	446	38 (9.5)		1.1 (0.5)	453	85 (17.5)		1.3 (0.7)
30–39 years	409	62 (16.8)		1.2 (0.7)	395	93 (22.4)		1.4 (0.7)
40–49 years	345	70 (20.9)		1.4 (0.8)	343	93 (25.4)		1.5 (0.8)
Residence			0.21				0.04*	
Rural	627	91 (17.3)		1.2 (0.6)	621	119 (17.2)		1.3 (0.7)
Urban	573	79 (12.9)		1.2 (0.7)	570	152 (26.1)		1.4 (0.9)
Marital status			0.009*				0.09	
Single	326	28 (9.8)		1.1 (0.5)	249	55 (23.6)		1.3 (0.6)
Married	854	136 (16.6)		1.3 (0.7)	873	191 (19.4)		1.4 (0.8)
Separate/ divorced/widow	20	6 (28.1)		1.4 (0.9)	69	25 (35.7)		1.7 (0.7)
Years of schooling^**b**^			0.03*				0.002*	
> 11 years	200	23 (9.8)		1.1 (0.5)	181	31 (14.0)		1.2 (0.6)
6–11 years	602	75 (13.7)		1.2 (0.6)	527	101 (17.7)		1.3 (0.7)
≤5 years	398	72 (19.0)		1.4 (0.8)	483	139 (27.9)		1.5 (0.8)
Occupation^c^			0.29				0.43	
Dependent/ students/ housewife	56	3 (8.7)		1.2 (0.5)	553	131 (22.3)		1.4 (0.8)
Unskilled worker	597	95 (17.2)		1.3 (0.7)	159	42 (26.0)		1.5 (0.7)
Government/non-government staff	182	23 (14.8)		1.1 (0.5)	109	24 (18.9)		1.3 (0.7)
Small own business	365	49 (11.9)		1.2 (0.5)	370	74 (18.3)		1.3 (0.7)
Income level (USD)^d^			0.001**				0.32	
*>* 3.1	186	16 (7.6)		1.1 (0.3)	286	51 (17.3)		1.3 (0.6)
1.9–3.09	260	29 (9.1)		1.2 (0.6)	318	72 (21.2)		1.3 (0.8)
< 1.9	752	125 (19.3)		1.3 (0.8)	584	148 (23.3)		1.4 (0.7)
Number of children			0.001**				0.001**	
No child	162	19 (11.2)		1.1 (0.4)	112	17 (10.4)		1.3 (0.7)
1–4 children	501	66 (13.3)		1.2 (0.7)	552	102 (15.3)		1.3 (0.7)
> 5 children	211	57 (29.8)		1.6 (0.8)	278	97 (34.5)		1.6 (0.7)
Migration			0.03*				0.04*	
No	1071	148 (14.0)		1.2 (0.7)	1038	225 (20.2)		1.4 (0.8)
Yes	129	22 (21.2)		1.4 (0.7)	153	46 (27.9)		1.5 (0.8)
Family member			0.001**				0.01*	
< 4	682	75 (11.2)		1.1 (0.5)	699	138 (17.5)		1.3 (0.7)
> 4	518	95 (20.1)		1.3 (0.8)	492	133 (26.8)		1.4 (0.8)
Household Debt			0.008*				0.001**	
No	517	58 (11.2)		1.1 (0.5)	476	75 (14.8)		1.2 (0.6)
Yes	679	111 (17.9)		1.3 (0.7)	713	196 (25.9)		1.5 (0.8)
General Health Status			0.004*				0.001**	
Good	868	98 (12.5)		1.1 (0.5)	627	108 (16.5)		1.2 (0.6)
Poor	332	72 (20.7)		1.4 (0.7)	564	163 (26.6)		1.5 (0.7)
Impairment^e^			0.007*				0.04*	
No	697	78 (11.6)		1.1 (0.5)	510	89 (17.6)		1.2 (0.6)
Yes	503	92 (19.4)		1.4 (0.7)	681	182 (24.0)		1.5 (0.7)
Musculoskeletal disorder			0.001**				0.001**	
no pain site	349	21 (5.8)		1.1 (0.4)	127	18 (10.4)		1 (0.3)
1–2 pain sites	564	80 (13.6)		1.2 (0.7)	610	111 (17.3)		1.3 (0.7)
3–5 pain sites	287	69 (27.2)		1.5 (0.8)	454	142 (29.6)		1.5 (0.7)
Work loss day due to pain			0.13				0.02*	
No	96	11 (11.2)		1.1 (0.4)	60	6 (9.7)		1.1 (0.3)
Yes	755	138 (18.9)		1.3 (0.8)	1004	247 (23.4)		1.4 (0.7)
Experienced traumatic event			0.05*				0.001**	
No	859	108 (13.1)		1.2 (0.6)	970	200 (19.1)		1.3 (0.7)
Yes	341	62 (18.9)		1.3 (0.8)	221	71 (30.5)		1.5 (0.8)
Domestic violence			0.002*				0.001**	
No	624	71 (11.4)		1.1 (0.4)	513	71 (14.3)		1.2 (0.6)
Yes	576	99 (18.4)		1.4 (0.7)	678	200 (26.6)		1.5 (0.8)

^a^Mental distress: > 1.85 HSCL score

^b^Years at school: < 5 years (primary education); 6–11 years (secondary education: middle and high schools); > 11 years (tertiary education: university)

^c^Occupation: Dependent (students, housewives and unable to work); Unskilled workers (odds job); Government and

nongovernment staff (getting monthly salary)

^d^Exchange rate: US$1 = 1363 Myanmar Kyats as of 4 November 2018

^e^Impairment of mobility/hearing/vision/washing/concentration. A positive answer in any of these symptoms is noted as ‘Yes’

*p < 0.05, **p < 0.001

Bivariate analysis showed that the prevalence of mental distress increased from the lowest age group (18–29 years: men: 9.5%; women: 17.5%) to the highest age group (40–49 years: men: 20.9%; women: 25.4%) (Table1). For both sexes, participants with less than five years of schooling demonstrated higher prevalence of mental distress than those with more than 11 years of schooling: 19% in men, (p = 0.03) and 27.9% in women, (*p* = 0.002). Moreover, we found higher prevalence of mental distress in divorced or widowed compared to married respondents. Similarly, low income compared to high income, unskilled workers compared to small own business worker showed increased mental distress prevalence. Factors such as increasing numbers of children, debt, domestic violence, and experience of a traumatic event were associated with higher mental distress. Moreover, health related factors, including poor self-reported health status, presence of any impairment, and multiple pain sites was also associated with mental distress (Table 1). In separate analyses by sex, we found a significant association between income and mental distress in men but not in women (Table 1). The highest median distress scores was found in women in the subgroup of separated/divorced/widowed (1.7; IQR: 0.7) and in the group who reported more than four children (1.6; IQR: 0.7) (Table 1). In men, the highest score was found among those having more than four children (1.6; IQR: 0.8) (Table 1).

In multivariable linear regression analyses (Table 2: Model 2), educational attainment (years at school) showed a significant negative association with mental distress after adjustment for age, marital status and income. The coefficients show that a one-year increase in education attainment, gives a 0.021 point decrease in HSCL score in women and a 0.012 point decrease in men, representing the total effects of education on mental distress. A significant interaction between education and age was observed in men. We assessed the association between years at school and mental distress at both young (18 years) and older age (49 years). We found a significant negative association between education and mental distress among older men but not among younger, indicating a 0.032 points decrease in HSCL score for a 1 year increase in years at school among older men (Table 2: Model 3b). Minor deviations from linearity were detected with the regression models identifying increasing error variances; however, using robust variance estimations did not change the result.

**Table 2:** Association^a^ between education and mental distress among 18–49-year-old citizens of Yangon Region, Myanmar

		Men		Women	
Model	Description	Coef (95% CI)	p-value	Coef (95% CI)	*p*-value
1	Crude	−0.014 (−0.023, −0.006)	0.002*	−0.023 (−0.032, −0.014)	0.000**
2	Adjusted^b^	−0.012 (−0.021, −0.004)	0.005*	−0.021 (−0.031, −0.011)	0.000**
3a	Adjusted with interaction term^c^, low age (18 years)	0.007 (−0.003, 0.016)	0.17		
3b	Adjusted with interaction term^c^, high age (49 years)	− 0.032 (− 0.04, − 0.02)	0.000**		

^a^Linear regression; ^b^Adjusted for age, income, marital status; ^c^Adjusted for age, income, marital status, interaction term (educ*age)

*p < 0.05, **p < 0.001

## Discussion

One in five females aged 18–49 years suffered from mental distress in the Yangon Region, Myanmar, while the prevalence was lower among men at 15%. After adjustment for confounders, an association between years at school and mental distress was found among women and the oldest men, but not among the youngest men (interaction). Crude analyses showed a high prevalence of mental distress in several subgroups related to socio-demographic and health factors, including low income and education, high number of children, domestic violence and poor self-perceived health status.

As far as the authors are aware, this is the first population-based study of mental distress in Myanmar. The strength of the present study is the high response rates, reducing errors from selection bias. We used internationally accepted and widely used questionnaires such as the HSCL-10 for mental distress and a modified version of the WHO DHS Myanmar questionnaire. The latter was already translated to Burmese [26], while the former was translated from English to Burmese and back translated, then piloted and adjusted to reduce the risk of information bias. Furthermore, the mental distress instrument has been validated in an Asian population (Pakistan) [28]. Another strength is that we assessed a one-week period-prevalence of mental distress, minimizing recall bias, compared to studies of one-month period-prevalence.

Compared with other countries, the prevalence of mental distress in the present study was lower than reported in Canada [15], Malaysia [14], Cambodia [11] and Kashmir Valley in India [35]. The longitudinal community survey from Canada [11] which included 2433 men and women aged 15 to 65 years used the K-10 scale for mental distress while the Malaysian National Health and Morbidity Survey (2015) [14] used the General Health Questionnaires (GHQ-12) in a sample of individuals older than 15 years. The Cambodia study [15] and the Kashmir study [35] applied the Hopkins Symptoms Checklist-25 (HSCL-25) in adult populations. Variations in design, instruments and/or included ages across studies make comparison of prevalence of mental distress difficult. Moreover, a study among Karenni (Myanmarnese) refugees in a Thai-Burmese border camp estimated that the prevalence rate of depression and anxiety were 42 and 41%, respectively, when measured with the HSCL-25 [36]. However, refugees may have been exposed to more trauma than a general population. In contrast, we found a higher prevalence of mental distress than reported in studies from Thailand [13], Vietnam [12], and Norway [16]. The Thai National Mental Health survey in 2013 used Composite International Diagnostic Interview Version 3.0 (CIDI 3.0) [13] among those older than 17 years. A population-based cross-sectional study among adults older than 15 years from Vietnam was based on the Self-Reporting Questionnaires (SRQ-20) which consists of a depression scale, an anxiety scale and a somatization scale [12]. The only study that used the same tools as in the present study was the study from Norway [13]. Prevalence estimates may, thus, vary between studies due to the different designs and tools used for screening, the varying periods of time of the studies, the different ages of participants and the use of different cut-off points for distress. Differences may also be attributed to various socio-cultural factors.

We report a higher prevalence of mental distress among women than men, which is in line with several other studies undertaken in other countries [11–15, 35, 37–39]. It is possible that women are more vulnerable to the socio-cultural risks, for example, a cultural belief that men have higher status relative to women, which have led to more depression among women [35, 37, 38]. In Myanmar, men have a dominated role as head of household, primary breadwinner and legal representative of the family [39]. Furthermore, women carry a heavier burden of household responsibility than men and that may predispose them to an increased risk of mental distress, for example, when the household has economic problems [35, 38]. Hormonal differences between sexes has also been suggested as being one of the contributing factors of higher prevalence of mental distress in women when compared with men [40, 41]. Moreover, mental distress was found to be predominantly higher in socially disadvantaged groups (low education, unemployed, those living in poverty), and among separated and divorced persons in the present study. These findings are consistent with previous studies in developed and developing countries [4, 11–15, 35, 37, 38].

Our findings of a negative association between years at school and mental distress amongst women, and men in the highest age groups (49 years), after controlling for confounding factors including age, income and marital status are in agreement with a household survey in Chile [42]. Education showed an inverse association with common mental health problems after adjusting for other socioeconomic variables [42]. Studies from India [35] and Pakistan [38] demonstrated that low educational attainment was highly associated with mental distress. By contrast, in a population-based Swedish study measured with the 12-item version of the General Health Questionnaire (GHQ), low educational level was not associated with mental distress after adjusting for confounding variables, but they did not check for interactions [23].

According to a Danish population-based follow up study of 16–29 year olds measuring the 12-item Short-Form Health Survey revealed that mental distress was significantly associated with students who fail to complete school [43], which may support the findings of the present study. In Myanmar, the enrolment rates after primary school drops sharply, indicating that a large proportion of young people end their educational pathway before reaching the upper secondary level [44]. There are several reasons for not completing school including parents unawareness of the value of education, school being too far away from home, poverty and inability to send their children to school, and lack of interest in keeping their children in an educational system [44]. One fifth of children in Myanmar aged 10–17 years were identified as being in some form of employment [44].

Studies have reported that characteristics in early life, such as poor health in childhood, have led to premature termination of schooling and worsening mental health condition in adulthood [43, 45]. An adolescent cohort study revealed that for participants with common mental health problems of duration less than 6 months at baseline, the symptoms may persist throughout adulthood [21], which makes continued schooling a challenge. The same study reported that adolescents who experienced parental separation or divorce had a higher risk of carrying ongoing common mental disorders from adolescence into young adulthood [21]. There is an intergeneration transmission between parental socioeconomic status and childhood and adult educational attainment [46]. Parental socioeconomic status, especially parental education and income, is one of the well-known factors to be associated with educational attainment of offspring, which also affects their mental health [47].

The Myanmar Health Statistics Report (2014–16) [48] using data from the Health Information and Management System (HMIS) from public health services in all states and regions of Myanmar revealed that six and seven persons per 100,000 of the population suffered from depression and anxiety, respectively, in 2016. The findings are under-reported as this system identifies only a small proportion of those affected. As a result, policy makers may use the data to denote that the prevalence of mental health problems amongst the population is very small and does not need to be prioritized.

In Myanmar, mental health care is limited, with only two psychiatric hospitals, which are located in Yangon and Mandalay townships. In other townships, the mental health services are provided in the general hospitals and community-based services are provided in rural health centers [10]. Around 2 % of the country’s national budget is spent on health, and less than 1% of it is spent on mental health services [10]. Besides, the mental health policy in Myanmar was last revised in 1995 and is still a part of the general health policy. The mental health legislation was enacted in 1912 and does not reflect international standards. Due to this, a new legislation has been drafted; however, it is still awaiting approval and enactment [10]. Reliable base-line data on the occurrence of mental health problems is needed in order to plan a healthy mental health policy in Myanmar.

A limitation of this study might be that we lack data on some potential confounders such as a family history of mental illness, parental educational and income level, genetic factors and childhood characteristics, resulting in the possibility of some residual confounding. Furthermore, a limitation might be the exclusion criteria, which excludes soldiers, institutionalized people, monks, nuns and those who are physically or mentally too ill to participate from the sampling frame. However, people were not excluded due to sickness in the study. Regarding the exclusion of soldiers, institutionalized people, monks and nuns, it is not known if this selection has led to an overestimation or underestimation of the prevalence of mental distress. We do not have information about the prevalence of mental distress or anxiety symptoms among soldiers, but it can be speculated that it is higher than a general population due to traumatic experiences from armed conflicts [49].

Information bias may arise when some categories of respondents fail to report their distress symptoms or complying with what the respondents feel is socially desirable [50], leading to underreporting. Another limitation is that the instrument for mental distress, HSCL-10, was not validated in a Myanmar population. Although the instrument has been validated in a Pakistani and Norwegian population, we cannot rule out if the symptoms of mental distress in the present study are over- or underreported. However, we have no reason to believe that any subgroups of our study-population responded differently to the HSCL-10 instrument due to lack of validation. Until a validation study has been performed, the results need to be interpreted with caution, although the standard English version of the mental distress questionnaires was translated by a psychiatrist to Burmese language and back translated, and a pilot study was conducted, resulting in minor corrections. A final limitation of the association measures is the cross-sectional design of the study. It is impossible to establish the direction of the association between years at school and mental distress, and thus, one cannot draw a conclusion about cause and effect. Therefore, further prospective studies are required.

## Conclusions

We have identified that a large segment of the adult population of Myanmar suffers from mental distress. It appears to be more common in socially disadvantaged groups (low education, unemployed, living in poverty), among women and among those with poor health status. There is an inverse association between educational attainment and mental distress in women and older adult men. Hence, the findings from this study provide baseline information about mental distress, contributing to increased awareness among health professional and policy makers, and giving a rationale for planning and implementation of mental health promotion and prevention campaigns in a community setting. Our findings will fill a knowledge gap regarding the occurrence of common mental health problems (mental distress) in the South Asian Region. We recommend further research on risk factors for mental distress using a longitudinal design.

## Data Availability

Data will be available upon request from the correspondence author.

## Abbreviations

DAGs: Directed acyclic graphs
DALYs: Disability-adjusted life-years
DHS: Demography and health survey
HMIS: Health Information and management system
HSCL: Hopkins symptom checklist
YLDs: Years lived with disability
